# Multi-mechanism synergistic remediation of phosphate-solubilizing bacteria under tetracycline and lead co-contamination stress: phosphate precipitation and organic acid complexation

**DOI:** 10.3389/fmicb.2025.1744505

**Published:** 2026-01-06

**Authors:** Yanjie Zhou, Jiangyan Wu, Qi Tang, Huihui Chen, Yuan Liu, Haoming Chen

**Affiliations:** 1 School of Civil Engineering, Southeast University, Nanjing, China; 2 School of Environmental and Biological Engineering, Nanjing University of Science and Technology, Nanjing, China; 3 Nanjing Institute of Geography and Limnology, Chinese Academy of Sciences, Nangjing, China

**Keywords:** co-contamination, microbial remediation, organic acidss, phosphate precipitation, synergistic effect

## Abstract

Tetracycline (TC) and lead (Pb) contamination have become globally urgent environmental challenges. They are widely distributed in soil, water bodies, and other environments, posing severe threats to ecosystems and human health. Microbial remediation, as a cost-effective and environmentally friendly pollution control approach, has garnered increasing attention in recent years. This study isolated and screened a highly efficient phosphate-solubilizing bacteria strain CZ-M3 (*Microbacterium* sp.) from a chemical factory contaminated environment. The strain achieved a phosphate solubilization capacity of 125.46 mg/L in PVK medium, significantly outperforming the control strain CZ-B5 (69.1% increase). Stress tolerance experiments demonstrated that strain CZ-M3 maintained robust activity under TC (≤200 mg/L) and Pb^2+^ (≤1,000 mg/L) stress, achieving 72-h removal rates of 57.36% for 200 mg/L TC and 28.5% for 1,000 mg/L Pb^2+^, thus selected as the core functional strain for remediating TC-Pb co-contamination. Notably, under co-contamination conditions, TC stress was found to stimulate Pb immobilization by CZ-M3. First, the strain releases PO₄^3−^ by secreting various organic acids, forming stable Pb-phosphate precipitates [e.g., Pb₅ (PO₄)₃Cl] with Pb^2+^, which was confirmed by X-ray diffraction (XRD) and attenuated total reflectance infrared spectroscopy (ATR-IR) results. Second, three-dimensional excitation-emission matrix (3D-EEM) spectroscopy revealed that combined stress induced enhanced secretion of extracellular polymeric substances (EPS) (humic acid-like substances), whose abundant functional groups (e.g., carboxyl and hydroxyl groups) effectively complexed and adsorbed contaminants. Most critically, organic acid secretion profiling found that high-concentration TC stress inhibited the tricarboxylic acid (TCA) cycle, causing a significant increase in tartaric acid content (significantly higher than the control group). Tartaric acid formed stable complexes with Pb^2+^ via its functional groups and promoted phosphate precipitation. This study provides a solid theoretical basis and a valuable microbial resources for the bioremediation of heavy metal-antibiotic co-contaminated environments.

## 1 Introduction

With global urbanization and agricultural development, persistent toxic pollutants such as antibiotics and heavy metals are causing irreversible harm to the environment and human health. Among these, the co-contamination of tetracycline (TC) and lead (Pb) represents a particularly challenging issue due to their synergistic toxic effects. According to statistical data, the global annual consumption of antibiotics is approximately 1 ~ 2 × 10^5^ tons (Li et al., 2023). TC, a broad-spectrum antibiotic, is widely used in healthcare, livestock breeding, and agriculture, with its stable chemical structure enabling persistent environmental retention (Li et al., 2021; Guo et al., 2023). Over 70% of TC is released into the environment via human and animal feces, which causes severe contamination (Daghrir and Drogui, 2013; Duong et al., 2024). Meanwhile, Pb is another prevalent persistent toxic pollutant characterized by high toxicity, bioaccumulation, and persistence (Shi et al., 2023; Ur Rahman et al., 2024). Critically, these pollutants often coexist in agricultural environments, where their interactions may lead to more complex contamination scenarios (Yu et al., 2022). For instance, antibiotics can form stable complexes with heavy metals through their functional groups, altering metal bioavailability and environmental behavior (Wan et al., 2010; Lykhin et al., 2014). This interplay necessitates the development of innovative remediation strategies capable of addressing both pollutants simultaneously.

Bioremediation, by virtue of its high efficiency, environmental friendliness, and sustainability, has become one of the core technologies for environmental pollution remediation. Bioremediation serves as a sustainable alternative to traditional methods such as chemical precipitation, ion exchange, chemical oxidation, and adsorption (Sedlakova-Kadukova, 2022). Particularly, microbial remediation technology has been proven effective in remediating antibiotic and heavy metal contamination (Yang et al., 2022; Shu et al., 2025). Microorganisms combat co-contamination through multiple mechanisms, including cell surface adsorption, intracellular accumulation, enzymatic transformation, and bioflocculation. These mechanisms not only remove or reduce contaminant toxicity from the environment but also mitigate the cytotoxicity of pollutants to microorganisms, thereby alleviating cellular damage (Wang et al., 2022; Radojević et al., 2023; Gul et al., 2024). However, current research on microbial tolerance and remediation mechanisms under TC-Pb co-contamination remains insufficient, and the development of highly efficient and broad-spectrum functional microbial inoculants remains challenging.

Phosphate-solubilizing microorganisms (PSM) are functional microbial groups capable of converting insoluble phosphorus into soluble phosphorus through metabolic activities (Chen et al., 2019; Rawat et al., 2021). Their core function is to secrete active substances such as organic acids, protons (H^+^), and phosphatases to dissolve inorganic phosphorus (e.g., calcium phosphate, apatite) or mineralize organic phosphorus (e.g., nucleic acids, phospholipids, phosphosugars), thereby releasing phosphate ions (PO₄^3−^) utilizable by plants or microorganisms (Chen et al., 2023a; Nassef et al., 2025). Beyond their agronomic importance, PSM have shown great potential in environmental remediation. PSM enhance TC hydrolysis by releasing organic acids to increase acidity and PO₄^3−^ (Tan et al., 2023; Lv et al., 2025). Simultaneously, PSM utilize extracellular proteins secreted by themselves to adsorb heavy metal ions, and generate PO₄^3−^ through organic acids and bioenzymes to form mineral precipitates with heavy metal ions (Li et al., 2018; Hu and Chen, 2023). However, the remediation mechanisms of PSM under TC-Pb co-contamination stress, particularly the metabolic adaptations (e.g., shifts in organic acid secretion profiles) and synergistic effects between phosphate precipitation and organic acid complexation, remain in the exploratory stage. A key unresolved question is how the metabolic network of PSM is altered under combined stress to drive synergistic remediation.

This study aims to screen phosphate-solubilizing bacteria with high-efficiency remediation capabilities for TC and Pb co-contamination, and to elucidate their TC degradation mechanisms and Pb immobilization mechanisms under co-contaminated conditions. This research will provide a theoretical basis for developing bioremediation technologies targeting TC-Pb co-contamination, contributing to the optimization of environmental remediation strategies and enhancement of remediation efficiency.

## 2 Materials and methods

### 2.1 Isolation and identification of phosphate-solubilizing bacteria

Phosphate-solubilizing bacteria were isolated from contaminated soil collected from a pesticide factory. One gram of soil sample was added to a conical flask containing LB liquid medium (10 g/L tryptone; 5 g/L yeast extract; 10 g/L sodium chloride) and incubated at 36 °C with shaking at 180 rpm for 72 h. 1 mL bacterial suspension was serially diluted with sterile saline, then inoculated onto LB solid medium (supplemented with 20 g/L agar) using the spread plate method, which was followed by aerobic incubation at 36 °C for 48 h. Distinct single colonies were selected and purified via streak plate method (identical conditions), yielding two bacterial strains: CZ-M3 and CZ-B5. Purified strains were identified by 16S rRNA gene sequencing.

### 2.2 Screening of PSB with optimal P-solubilizing capacity and stress tolerance

(1) Verification of phosphate-solubilizing function

This study employed the liquid phosphate-solubilizing method to verify the phosphate-solubilizing function of CZ-M3 and CZ-B5. 2 mL bacterial suspension was added to 100 mL PVK liquid medium and NBRIP liquid medium, respectively, followed by shaking incubation at 30 °C and 180 rpm for 72 h. The culture broth at different time points (1, 3, 6, 12, 24, 48, 72 h) was centrifuged and filtered (5,000 rpm, 0.22 μm). The soluble phosphorus content in the supernatant (subtracting the medium blank value, unit: mg/L) was then measured using Inductively Coupled Plasma Optical Emission Spectrometry (ICP-OES). PVK medium contained glucose 10 g, (NH₄)₂SO₄ 0.5 g, NaCl 0.3 g, KCl 0.3 g, FeSO₄·7H₂O 0.3 g, MgSO₄·7H₂O 0.3 g, MnSO₄·4H₂O 0.03 g, yeast extract 0.5 g, Ca₃ (PO₄)₂ 5 g, and was adjusted to 1 L with deionized water. NBRIP medium consists of glucose 10 g, Ca₃ (PO₄)₂ 5 g, MgCl₂·6H₂O 5 g, MgSO₄·7H₂O 0.25 g, KCl 0.2 g, (NH₄)₂SO₄ 0.1 g, and was adjusted to 1 L with deionized water.

(2) TC and Pb^2+^ stress experiments

2 mL bacterial suspension was added to 100 mL LB medium containing different concentrations of TC (50, 100, 200 mg/L) or Pb^2+^ [500, 1,000, 2000, 3,000 mg/L, sourced from Pb(NO₃)₂]. The samples were then incubated at 35 °C and 180 rpm with shaking for 72 h. Optical density (OD₆₀₀) at different time points (1, 3, 6, 12, 24, 48, 72 h) was measured using a UV spectrophotometer (wavelength: 600 nm) to characterize bacterial growth. After cultivation, samples from different stress concentrations (72 h) were centrifuged and filtered (5,000 rpm, 0.22 μm membrane). TC concentration and Pb^2+^ concentration in the solution were determined by High Performance Liquid Chromatography (HPLC) and ICP-OES, respectively. Each treatment was performed in triplicate.

### 2.3 Investigation of growth characteristics of phosphate-solubilizing bacteria

This study screened strains for TC-Pb co-remediation based on optimal phosphate-solubilizing capacity and highest stress tolerance. 2 mL bacterial suspension of the selected optimal strain was added to 100 mL LB medium. The cultures were incubated at different temperatures (30, 35, 40 °C, pH = 7) and different initial pH (4, 5, 6, 7, 8, temperature = 35 °C) with shaking at 180 rpm for 72 h. Optical density (OD₆₀₀) at different time points (1, 3, 6, 12, 24, 48, 72 h) was measured to characterize bacterial growth. Each treatment was performed in triplicate.

### 2.4 Study on remediation mechanisms of phosphate-solubilizing bacteria for TC-Pb co-contamination

Based on strain screening and growth characteristic results (Sections 2.2 and 2.3), this study established three TC-Pb co-contamination treatments for remediation experiments. The concentration ratios of TC and Pb were 200 mg/L: 500 mg/L, 50 mg/L:500 mg/L, and 50 mg/L:1000 mg/L, respectively. 2 mL bacterial suspension was added to 100 mL LB solution containing mixed TC and Pb^2+^ stress, followed by shaking incubation at 35 °C and 180 rpm for 72 h. Bacterial growth (OD₆₀₀) was measured at different time points (1, 3, 6, 12, 24, 48, 72 h). Each treatment had three replicates, with no-stress treatment as control. All measured concentrations (mg/L) were corrected by subtracting the background values of the medium. Additionally, 72 h samples from different treatments were centrifuged (50 mL, 5,000 rpm, 5 min) for solid–liquid separation.

A portion of the liquid sample was used for 3D-EEM analysis, while another portion was filtered through a 0.22-μm membrane for subsequent determination of organic acids and TC concentration by HPLC, and Pb^2+^ content by ICP-OES. A portion of the solid sample was dried and used for ATR-IR, XRD, and electrochemical measurements. Another part was used for SEM scanning electron microscopy analysis. The solid sample was fixed with 2.5% glutaraldehyde for 24 h, and then washed three times with 0.1 mol/L phosphate buffer solution (pH 7.0) for 15 min per wash. Subsequently, the sample was dehydrated with an ethanol gradient (20, 50, 75, 80, 90, 99%, 10 min each) and then subjected to freeze-drying for measurement.

### 2.5 Instruments and testing methods

#### 2.5.1 Strain identification methods

Use FastDNA SPIN Kit (Bio Teke, Co., China) to extract genomic DNA (Zhou et al., 2018). Primers 27F (5 ′- AGA GTTTGATCMTGCTCAG-3 ′) and 1492R (5 ′- CRGYTACCTGTTACGA-3 ′) were used for PCR amplification of 16S rRNA gene (Biswas et al., 2018). The nucleotide sequences were compared by BLAST program, and then the phylogenetic tree was constructed by MEGA7.0 software (Zhang et al., 2023).

#### 2.5.2 Sample detection and analysis

The detection items for liquid samples include bacterial OD600, soluble total phosphorus, Pb^2+^, TC, organic acids (formic acid, oxalic acid, malic acid, succinic acid, tartaric acid, citric acid), and biological secretions in the solution. The relevant detection methods refer to previous studies (Zhang et al., 2023; Lv et al., 2025). The OD600 value of the bacterial solution was determined using a UV–visible spectrophotometer (752 NPlus, Shanghai Yidian, China). The concentrations of soluble total phosphorus and Pb^2+^ were determined by ICP-OES (icap7000, ThermoFisher Scientific Inc. iCAP PRO, USA), with samples being centrifuged (5,000 rpm, 5 min) and filtered through a 0.22 μm filter membrane prior to determination, and the standard curve was prepared using national standard solutions. The concentrations of TC and organic acids were determined by HPLC (U3000, ThermoFisher Scientific Inc., USA), with an Inertsustain AQ-C18 column (5 μm, 4.6 × 250 mm), an injection volume of 20 μL, a flow rate of 1 mL/min, a mobile phase of 0.25% KH_2_PO_4_ buffer solution and methanol (volume ratio 99:1, 2.5%, pH = 2.8), a column temperature of 30 °C, and a detection wavelength of 210 nm. Biological secretions were detected by three-dimensional excitation-emission matrix (3D-EEM, F-7100, Hitachi, Japan), with excitation and emission wavelengths ranging from 250 to 800 nm and a diffraction slit width of 5 nm.

The detection of solid samples includes XRD, ATR-IR, SEM, and electrochemical detection. The relevant detection methods refer to previous studies (Chen et al., 2023b; Zhang et al., 2023). XRD (D8 Advance, Bruker AXS GMBH, Germany, Cu Kα, *λ* = 1.5406 Å; 40 kV; 40 mA; 2θ scanning range 10°-80°) was used to determine the mineral composition, and the types of minerals were confirmed by comparing with the PDF cards using the JADE 6.0 software. ATR-IR (Nicolet iS5 Fourier-transform infrared spectrometer, ThermoFisher Scientific Inc.) was used to analyze the functional group characteristics in the solid samples. The morphology and elemental composition of the solid samples were detected by SEM (Hitachi Regulus 8,100, Japan) and EDS (INCA 300 Oxford, UK). The electrochemical test was performed using CV with an electrochemical workstation (CHI760D, CHInstrument Company, China) on the dried samples, with carbon electrodes as the working and auxiliary electrodes and a saturated calomel electrode as the reference electrode.

## 3 Results and discussion

### 3.1 Isolation and identification of strains

**Figure 1:**
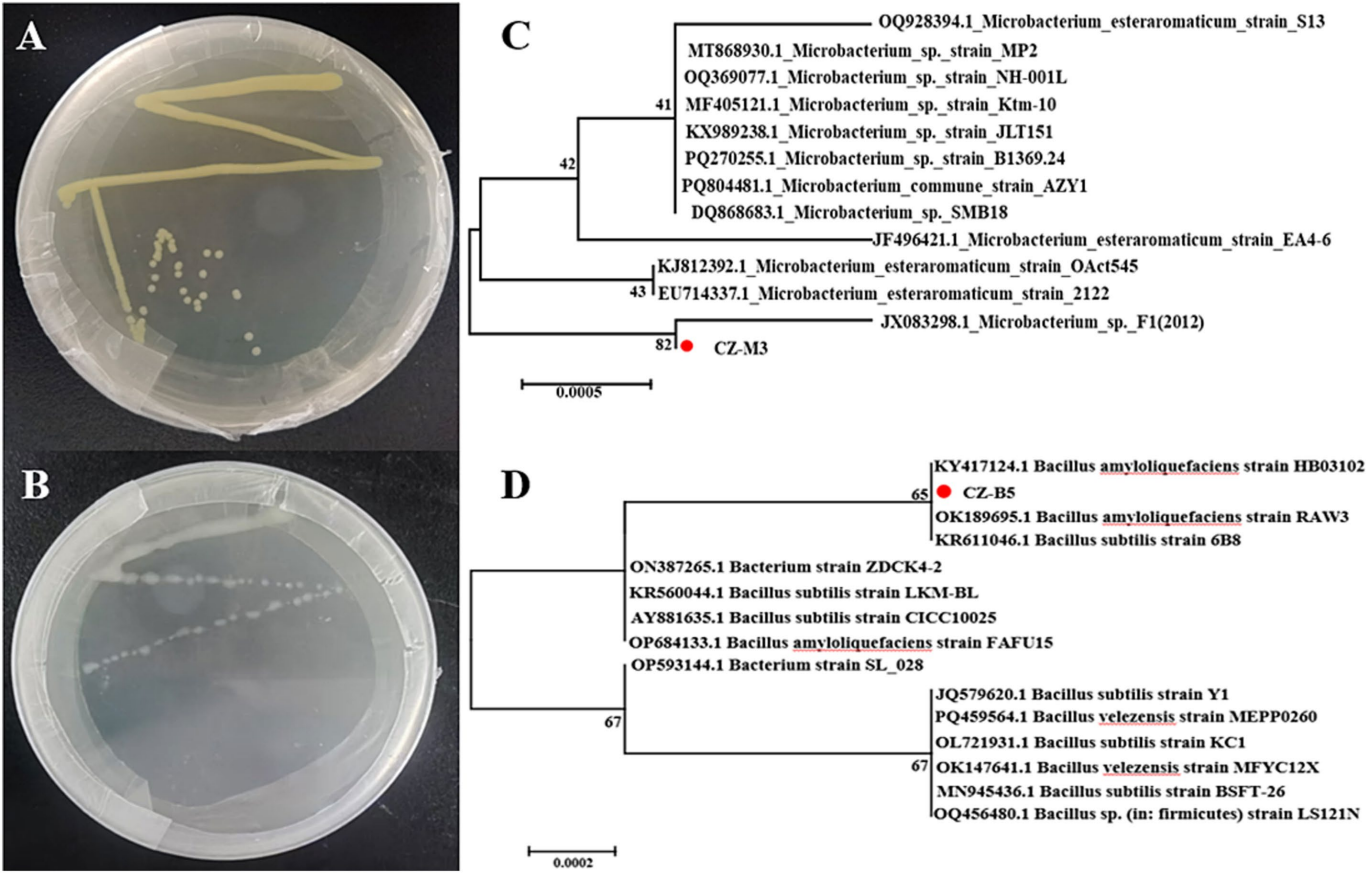
Growth conditions of bacteria CZ-M3 **(A,C)** and CZ-B5 **(B,D)** and the phylogenetic tree constructed based on the 16S rRNA sequences.

CZ-M3 and CZ-B5 were isolated and purified by streaking. Figures 1A,B show the colonies of CZ-M3 after 36 h and CZ-B5 after 12 h of cultivation, respectively. CZ-M3 exhibited relatively slow growth, reaching a robust growth state at 36 h with yellow colonies and no soluble pigments. In contrast, CZ-B5 grew rapidly, forming off-white or light yellow, rough, opaque, and uniformly colored colonies within 12 h. 16S rRNA gene amplification yielded fragments of approximately 1,403 bp for CZ-M3 and 1,430 bp for CZ-B5. Sequence analysis revealed that strain CZ-M3 shared the highest homology (99.86%) with *Microbacterium* sp. F1 (JX083298.1), and phylogenetic analysis placed them on the same branch (Figure 1C). Therefore, CZ-M3 was identified as a *Microbacterium* species. Strain CZ-B5 showed the highest homology (99.86%) with *Bacillus* sp. (OQ719134.1) and was its closest relative in the phylogenetic tree (Figure 1D), leading to its identification as *Bacillus subtilis*. Both strains have been deposited in the China General Microbiological Culture Collection Center (CGMCC) under accession numbers GCMCC 1.60034 (CZ-M3) and GCMCC 1.60035 (CZ-B5).

### 3.2 Phosphate solubilization and stress resistance of strains

#### 3.2.1 Phosphate solubilization effect

**Figure 2:**
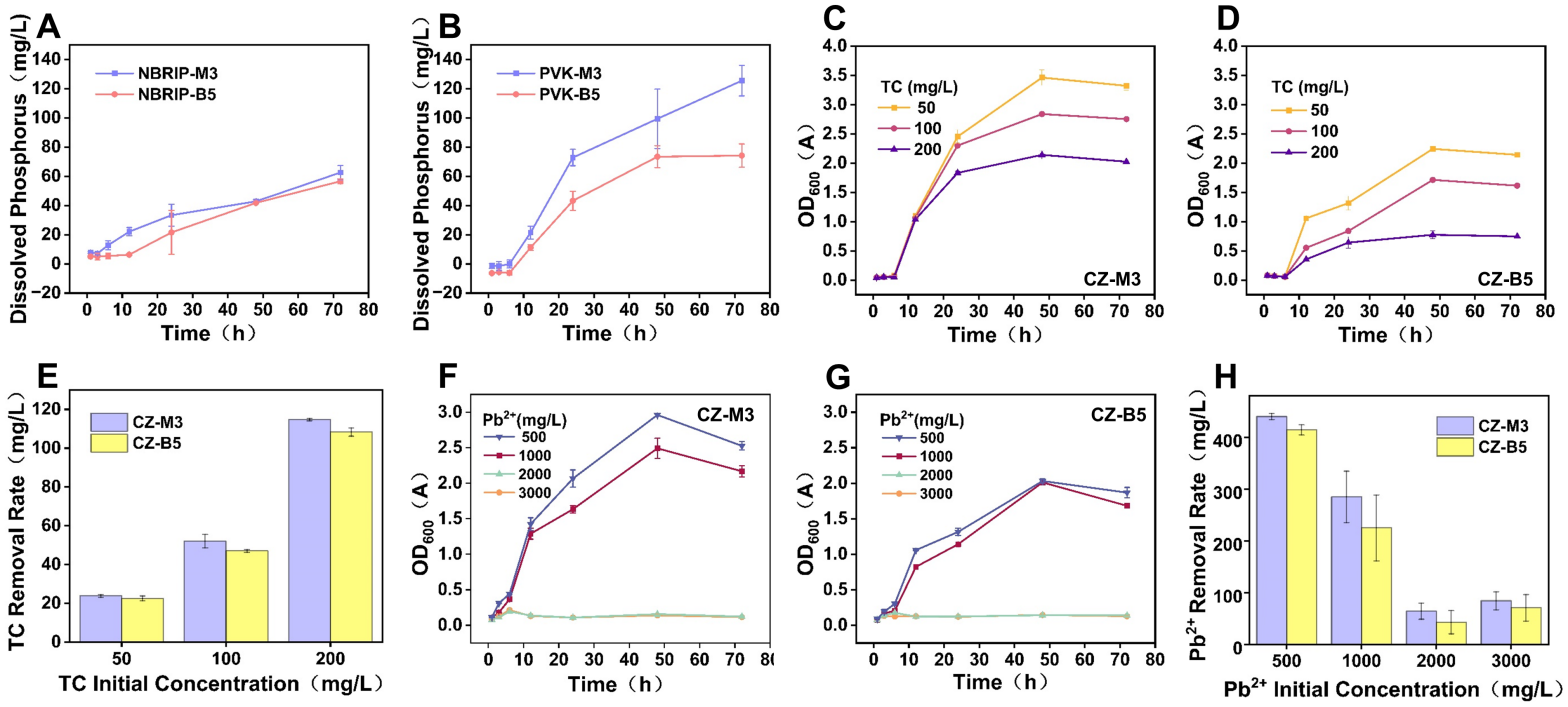
Phosphate solubilization by CZ-M3 and CZ-B5 and their growth and pollutant removal under stress (**A,B**: Phosphate solubilization of bacteria in NBRIP and PVK liquid media, respectively. **C,D**: Growth of CZ-M3 and CZ-B5 under TC stress. **E**: TC removal in solution after 72 h. **F,G**: Growth of CZ-M3 and CZ-B5 under Pb^2+^ stress. **H**: Pb^2+^ removal in solution after 72 h).

In the NBRIP medium system, both CZ-M3 and CZ-B5 exhibited phosphate solubilization kinetics that increased with time, and the two strains showed a similar trend in phosphate solubilization (Figure 2A). During the initial cultivation period (0–24 h), the phosphate solubilization rate of CZ-M3 and CZ-B5 increased significantly, followed by a slow-release phase. By the end of the 72-h cultivation, the difference in phosphate solubilization between the two strains was 5.98 mg/L. It is worth noting that when the medium system was switched to PVK (Figure 2B), a significant difference in phosphate solubilization efficiency was observed between the two strains. Compared with CZ-B5, CZ-M3 showed a stronger ability to activate phosphate. After 72 h of cultivation, the phosphate solubilization capacity of CZ-M3 was 69.1% higher than that of CZ-B5, and its phosphate solubilization kinetic curve consistently maintained a higher slope value. This phenomenon indicates that CZ-M3 has a stronger ability to dissolve insoluble phosphate sources.

#### 3.2.2 Stress resistance

(1) TC stress experiment

The results of the growth of strains under different concentrations of TC stress showed (Figures 2C,D) that when the TC concentration was ≤ 200 mg/L, both CZ-M3 and CZ-B5 maintained a good survival state, and the TC removal effect of CZ-M3 was better than that of CZ-B5. TC at a concentration of 200 mg/L had an inhibitory effect on the growth of both CZ-M3 and CZ-B5. The growth of CZ-M3 began to enter the logarithmic growth phase at 6 h and entered the stationary phase after 48 h. The growth trend of CZ-B5 was similar to that of CZ-M3, but its OD value was lower than that of CZ-M3 throughout the process, which confirmed that CZ-M3 had stronger TC resistance than CZ-B5. After 72 h of cultivation, the removal rates of TC (50, 100, and 200 mg/L) by CZ-M3 were 2.57, 4.99, and 3.21% higher, respectively, than those of CZ-B5 (Figure 2E).

(2) Pb^2+^ Stress Experiment

When the concentration of Pb^2+^ was ≤ 1,000 mg/L, both strains showed good tolerance, and their OD600 values gradually increased in the first 48 h (Figures 2F,G). When the concentration of Pb^2+^ reached 2000 mg/L, the OD600 values of CZ-M3 and CZ-B5 significantly decreased, and no recovery proliferation occurred in the later stage of culture, indicating that the concentration of 2000 mg/L Pb^2+^ exceeded the critical threshold of CZ-B5 and CZ-M3. It is worth noting that under the condition of Pb^2+^ concentration ≤ 1,000 mg/L, the OD600 value of CZ-M3 was significantly better than that of CZ-B5, indicating that CZ-M3 had stronger resistance to Pb^2+^ stress than CZ-B5. In addition, the removal rates of 500 mg/L and 1,000 mg/L Pb^2+^ stress by CZ-M3 were 5.8–6.0% higher than those of CZ-B5 (72 h) (Figure 2H). This phenomenon may be attributed to the increased toxicity of Pb, which severely inhibited bacterial reproduction and growth functions, thus interfering with their adsorption of Pb.

In summary, CZ-M3 demonstrated a 69.1% higher phosphate solubilization capacity in PVK medium and exhibited better growth and superior removal efficiency for both TC and Pb^2+^ under stress conditions, compared to CZ-B5. Therefore, CZ-M3 was selected as the core functional strain for remediating TC-Pb co-contamination for further investigation of its growth performance.

### 3.3 Growth characteristics of CZ-M3

**Figure 3:**
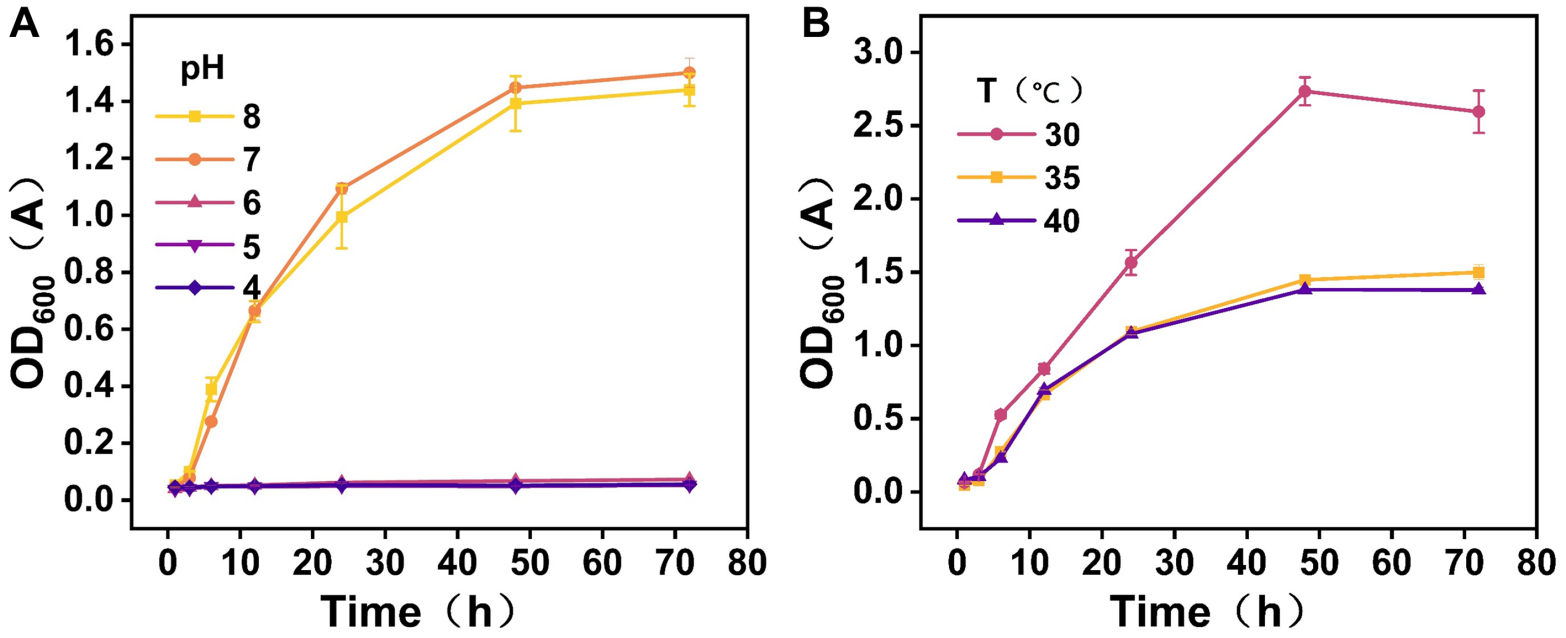
Growth curves of CZ-M3 at different pH **(A)** and temperatures **(B)**.

The OD600 value of CZ-M3 was highest at pH 7.0, indicating that this pH environment was most suitable for the growth of CZ-M3 (Figure 3A). When the pH of the system was increased to weakly alkaline (pH 8.0), the growth of CZ-M3 was only slightly inhibited (a decrease of approximately 4.4% compared to the neutral environment), suggesting that CZ-M3 can also survive in weakly alkaline conditions. Under more strongly alkaline conditions, significant metabolic disturbances occurred in CZ-M3, leading to growth inhibition. In acidic conditions (pH ≤ 6), CZ-M3 completely lost its proliferative capacity, indicating that it was not suitable for survival in acidic environments.

The growth curves of CZ-M3 at different temperatures are shown in Figure 3B. CZ-M3 exhibited optimal metabolic activity at 30 °C, with an OD600 value significantly higher than at other temperatures, indicating that this is the optimal growth temperature for the strain. Moreover, CZ-M3 maintained a good survival state within the temperature range of 30–40 °C. At 35–40 °C, the growth of CZ-M3 stabilized after 24 h, while at 30 °C, the growth of CZ-M3 began to decline after 48 h. The ability of CZ-M3 to maintain high activity between 30–40 °C indicates its good adaptability to temperature.

### 3.4 Remediation effect of CZ-M3 under co-contamination

**Figure 4:**
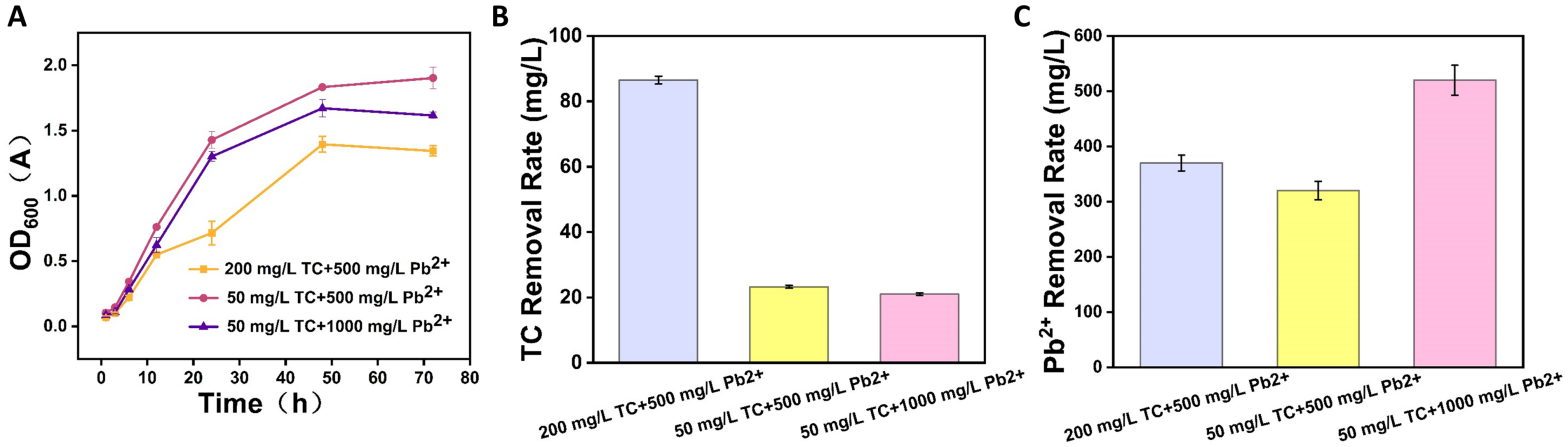
Growth curves of CZ-M3 under co-contamination **(A)**, TC removal after 72 h **(B)**, and Pb^2+^ removal after 72 h **(C)**.

Among the three TC-Pb co-contamination treatments, CZ-M3 showed the highest growth activity in the 50 mg/L TC + 500 mg/L Pb^2+^ group and the lowest in the 200 mg/L TC + 500 mg/L Pb^2+^ group (Figure 4A). In the low-stress group (50 mg/L TC + 500 mg/L Pb^2+^), the removal rates of TC and Pb^2+^ were 1.30 and 4.00% lower, respectively (Figures 4B,C), than those under the corresponding single-stress conditions (Figures 2E,H). This suggests that co-contamination exerts a stronger inhibitory effect on CZ-M3, potentially impairing its remediation function.

When the Pb^2+^ concentration was increased (50 mg/L TC + 1,000 mg/L Pb^2+^), the removal rates of TC and Pb^2+^ were 41.98 and 52.0%, respectively, indicating that high Pb^2+^ concentration significantly inhibited TC removal by the strain. In contrast, when the TC concentration was high (200 mg/L TC + 500 mg/L Pb^2+^), the removal rates were 43.3% for TC and 74.0% for Pb^2+^. Notably, the increase in TC appeared to stimulate the removal of Pb^2+^, whereas the increase in Pb^2+^ reduced the removal of both pollutants. This was likely because the higher Pb^2+^ concentration approached the tolerance limit of CZ-M3, thereby compromising its viability and remedial capacity.

### 3.5 Morphological characterization of CZ-M3 under co-contamination

**Figure 5:**
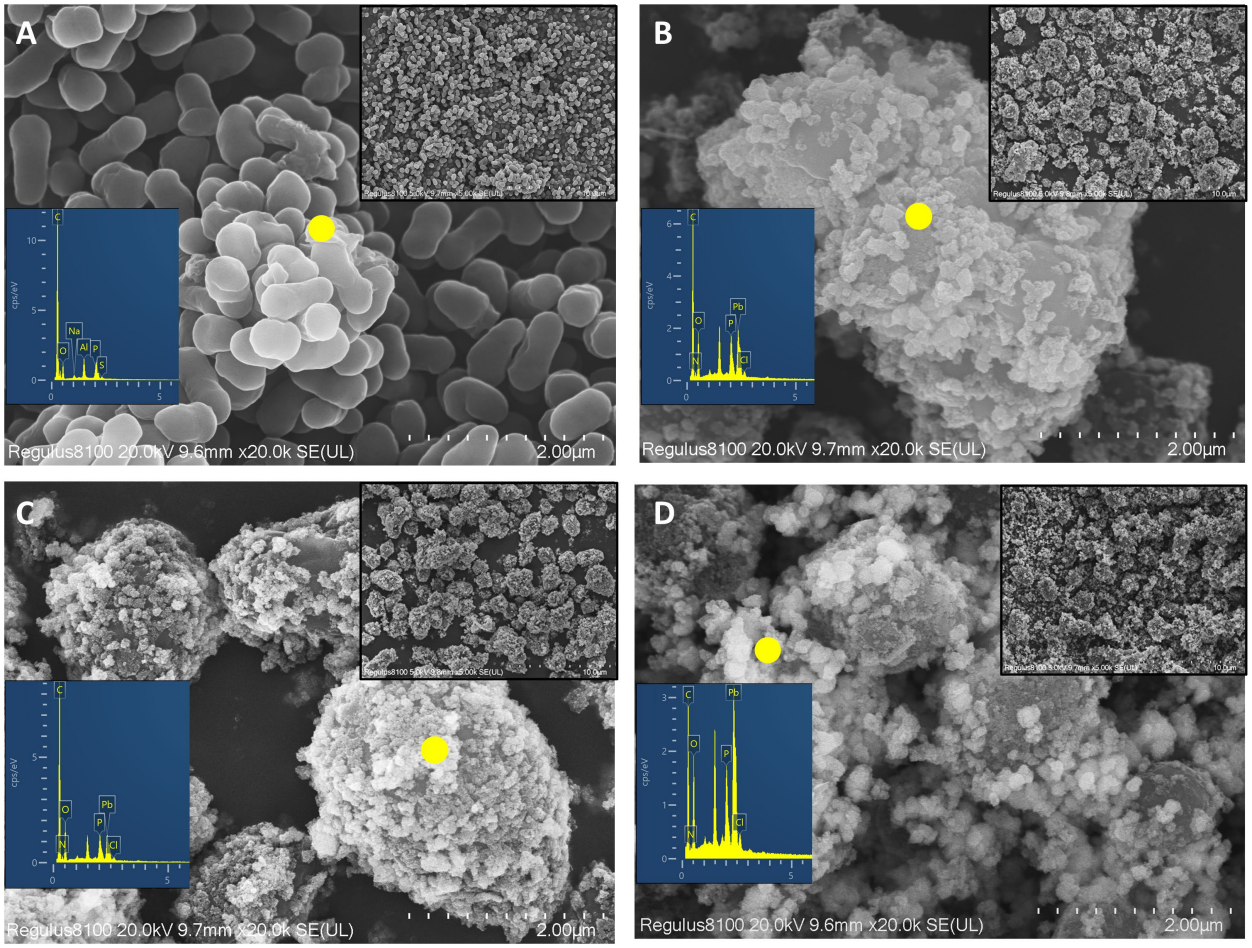
SEM and EDS of CZ-M3 after 72 h of co-contamination stress (**A**: No stress; **B**: 200 mg/L TC + 500 mg/L Pb^2+^ stress; **C**: 50 mg/L TC + 500 mg/L Pb^2+^ stress; **D**: 50 mg/L TC + 1,000 mg/L Pb^2+^ stress).

Scanning electron microscopy observations revealed that under no stress conditions, CZ-M3 cells appeared rod-shaped (Figure 5A) with a length of 0.6–1.0 μm and a smooth surface. Extracellular secretions were present around the cells of CZ-M3 after 72 h of cultivation. Under the culture condition of 200 mg/L TC + 500 mg/L Pb^2+^, the cells of CZ-M3 were enveloped by distinct crystals (Figure 5B), and the cells were deformed, indicating that the TC-Pb co-contamination hindered the growth and development of CZ-M3. In the group with 50 mg/L TC + 500 mg/L Pb^2+^, the cells of CZ-M3 were also enveloped by distinct crystals (Figure 5C), and the cells themselves were deformed. In the group with 50 mg/L TC + 1,000 mg/L Pb^2+^, there was a significant increase in crystals around CZ-M3 cells (Figure 5D), and the cell size was reduced by 0.3–0.5 μm compared with the previous two groups (200 mg/L TC + 500 mg/L Pb^2+^ and 50 mg/L TC + 500 mg/L Pb^2+^) (Figures 5B,C).

Under different TC and Pb concentrations, EDS analysis revealed differences in the remediation mechanisms of Pb by the phosphate-solubilizing bacterium CZ-M3 and its metabolic products. In the CK group without stress, C (79.71%) and O (17.17%) were the main components in the bacterial secretions, with a P content of 0.68%. In the group with 50 mg/L TC + 500 mg/L Pb^2+^, the contents of P (1.03%) and Pb (6.50%) increased compared with the CK group, suggesting that the stress of TC and Pb activated the adaptive response of CZ-M3. In the group with 200 mg/L TC + 500 mg/L Pb^2+^, the contents of P (1.46%) and Pb (10.44%) increased in the crystals enveloping the cells. In the group with 50 mg/L TC + 1,000 mg/L Pb^2+^, the Pb content increased significantly to 24.7%, and the contents of P (2.17%) and Cl (0.5%) also increased. CZ-M3 produced PO₄^3−^ by dissolving phosphates, which then combined with Pb^2+^ to form minerals. The increase in TC and Pb concentrations triggered stress responses in CZ-M3, thereby enhancing the release of secretions.

### 3.6 Characteristics of microbial secretions under co-contamination

#### 3.6.1 3D-EEM analysis

**Figure 6:**
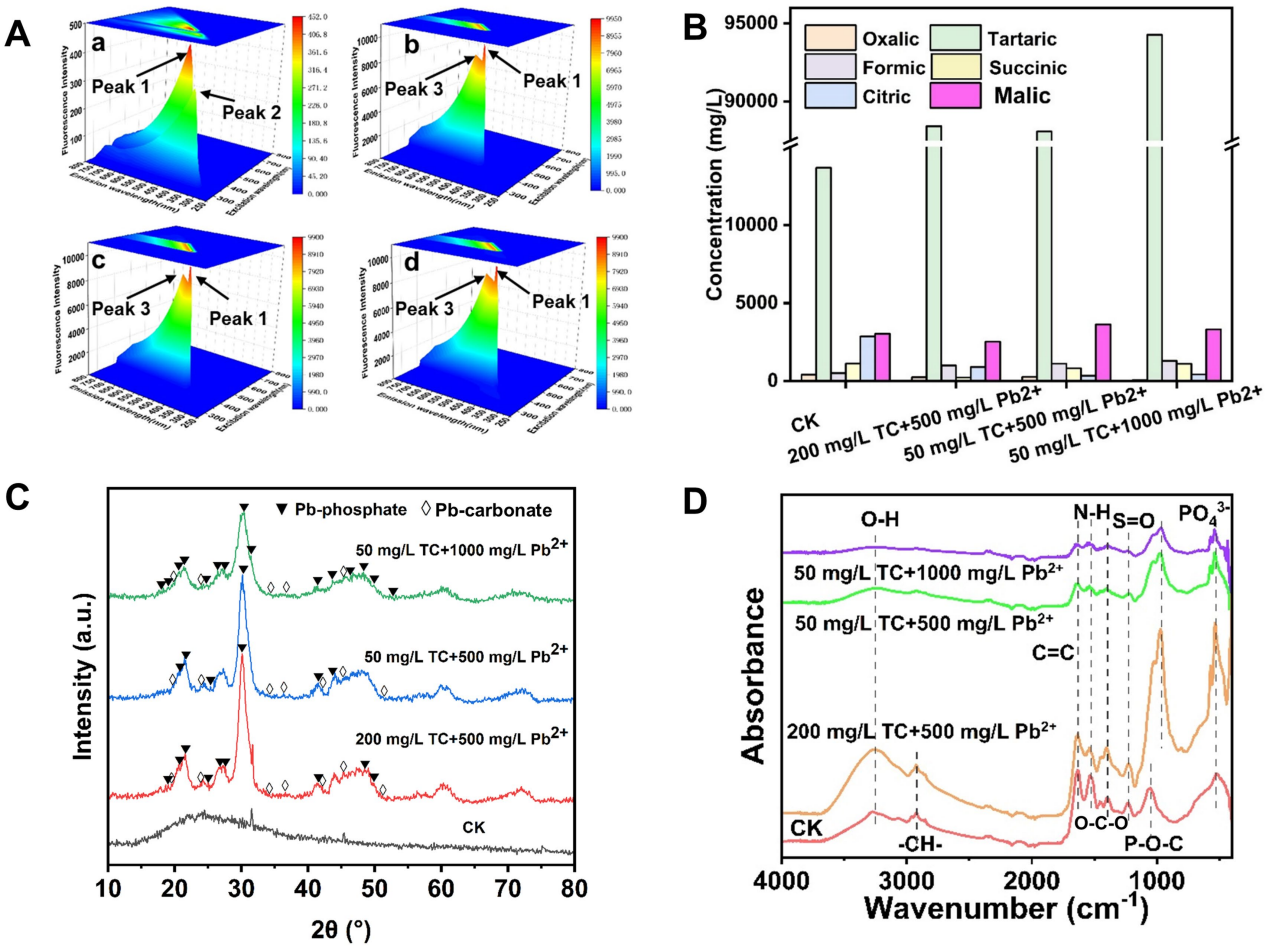
Results of 3D-EEM **(A)**, organic acids **(B)**, XRD **(C)**, and ATR-IR **(D)** of CZ-M3 after 72 h of co-contamination stress (a: No stress; b: 200 mg/L TC + 500 mg/L Pb^2+^ stress; c: 50 mg/L TC + 500 mg/L Pb^2+^ stress; d: 50 mg/L TC + 1,000 mg/L Pb^2+^ stress).

Under the stress of TC-Pb co-contamination, the metabolic response mechanism of CZ-M3 could be interpreted through the shift in peak positions and changes in intensity of its secretions as analyzed by 3D-EEM spectroscopy (Figure 6A). In the CK group, two characteristic peaks were primarily detected: peak 1 (Ex/Em = 455/435 nm, intensity 443.11) and peak 2 (Ex/Em = 390/370 nm, intensity 324.43) (Figure 6A), which corresponded to humic substances (Guo et al., 2014; Zhang et al., 2023). Under co-contamination, the fluorescence intensity of peak 1 significantly increased, with a slight shift in peak position (Ex/Em = 435–455/425–440 nm, intensity 9800.81–9939.6, Figures 6A–D). This change may have originated from the self-protection mechanism of CZ-M3, leading to the structural reconfiguration or an increased content of humic-like substances. Humic-like substances, which contain a large number of functional groups such as carboxyl and phenolic hydroxyl groups, can immobilize Pb^2+^ or TC through mechanisms such as electrostatic attraction, complexation, and ion exchange (Yang et al., 2021; Fu et al., 2023; Chen et al., 2024). The newly emerged peak 3 (Ex/Em = 520–525/500–505 nm, intensity 8552.13–8564.86) was likely a secretion produced by microorganisms under stress. Additionally, peak 2 completely disappeared, which may be due to the interference of the TC-Pb co-contamination stress with the normal metabolic pathways of bacteria.

#### 3.6.2 Organic acid secretion characteristics

The secretion of organic acids is crucial for phosphate-solubilizing bacteria in terms of phosphorus solubilization and heavy metal remediation. Under different concentrations of TC and Pb^2+^ stress, CZ-M3 secreted organic acids with significant differences (Figure 6B). In the stress-free group, higher concentrations of tartaric acid (13670.14 mg/L), citric acid (2862.73 mg/L), and malic acid (3035.13 mg/L) were detected (Figure 6B). Under mixed stress, the content of tartaric acid increased markedly, while that of citric acid decreased significantly, which may be attributed to the fact that high concentrations of TC may inhibit the activity of key enzymes in the tricarboxylic acid cycle (TCA), which blocked the synthesis pathway of citric acid and instead promoting the accumulation of tartaric acid. In addition, under a high concentration of TC (200 mg/L) stress, the decrease in succinic acid content was the most significant compared with that in the CK group, which may be due to the TC-induced blockage of the synthesis of the intermediate citric acid, a precursor to succinic acid.

Studies have shown that tartaric acid can form stable complexes with Pb^2+^ through carboxyl and hydroxyl functional groups and induce the release of phosphate to generate insoluble Pb-phosphate precipitates (Islam et al., 2022; Chen et al., 2023b). The significant increase in tartaric acid under a high concentration of Pb^2+^ (1,000 mg/L) also indicates that CZ-M3 increased tartaric acid secretion to counteract Pb^2+^ stress. Notably, a high concentration of Pb^2+^ (1,000 mg/L) led to a sharp decrease in oxalic acid content, which may be due to the Pb^2+^ stress-induced slowdown of cellular metabolism and reduction in the activity of CZ-M3. At the same time, oxalic acid may also be largely consumed by complexation with Pb^2+^ in the early stage of the reaction.

### 3.7 Characterization of heavy metal immobilization products under co-contamination

XRD results showed that Pb-phosphate and Pb-oxalate were present in the precipitates of all three treatment groups (Figure 6C). Among them, the group under 50 mg/L TC + 1,000 mg/L Pb^2+^ stress had the most characteristic peaks for Pb-phosphate and the fewest for Pb-oxalate. The group under 200 mg/L TC + 500 mg/L Pb^2+^ stress had the fewest characteristic peaks for both Pb-phosphate and Pb-oxalate. High concentrations of Pb^2+^ may trigger the stress resistance mechanism of CZ-M3, forcing the bacteria to preferentially combine the limited PO₄^3−^with Pb^2+^ to reduce external stress, resulting in an increase in Pb-phosphate. Higher concentrations of TC (200 mg/L) may inhibit bacterial activity, leading to a reduction in the secretion of organic acids (such as citric acid and oxalic acid) by CZ-M3, thereby limiting the formation of Pb-phosphate and Pb-oxalate.

Infrared results showed (Figure 6D) that in the CK group, there were peaks for C=C/N-H (1,634 cm^−1^ and 1,536 cm^−1^), -OH peaks (3,280 cm^−1^), and -CH₃/-CH₂ (2,975 cm^−1^) (Doshi et al., 2007; Wang et al., 2020), which may indicate the presence of hydroxylated lipids or sugars in extracellular polymeric substances (EPS) (Doshi et al., 2007; Wang et al., 2020). Under TC and Pb co-stress, the P-O vibrations at 538 cm^−1^ and 1,051 cm^−1^ suggested the presence of phosphorus-containing compounds, likely from lead phosphate (Chen et al., 2023a). Under 200 mg/L TC + 500 mg/L Pb^2+^ stress, the intensity of the -OH peak was significantly higher than that in CK, which may be due to the fact that high concentrations of TC-Pb stress may induce the bacteria to secrete more hydroxyl-containing EPS (such as polysaccharides or lipopolysaccharides) to mitigate toxicity. The ubiquitous symmetric stretching vibration of carboxylates at 1404 cm^−1^ indicates that the bacteria continuously secrete organic acids such as oxalic acid and citric acid to chelate Pb^2+^, increasing the spectral signals related to PO₄^3−^ (Zhang et al., 2023), confirming the presence of Pb-phosphate compounds in the precipitates.

### 3.8 Remediation mechanism of TC-Pb co-contamination by CZ-M3

**Figure 7:**
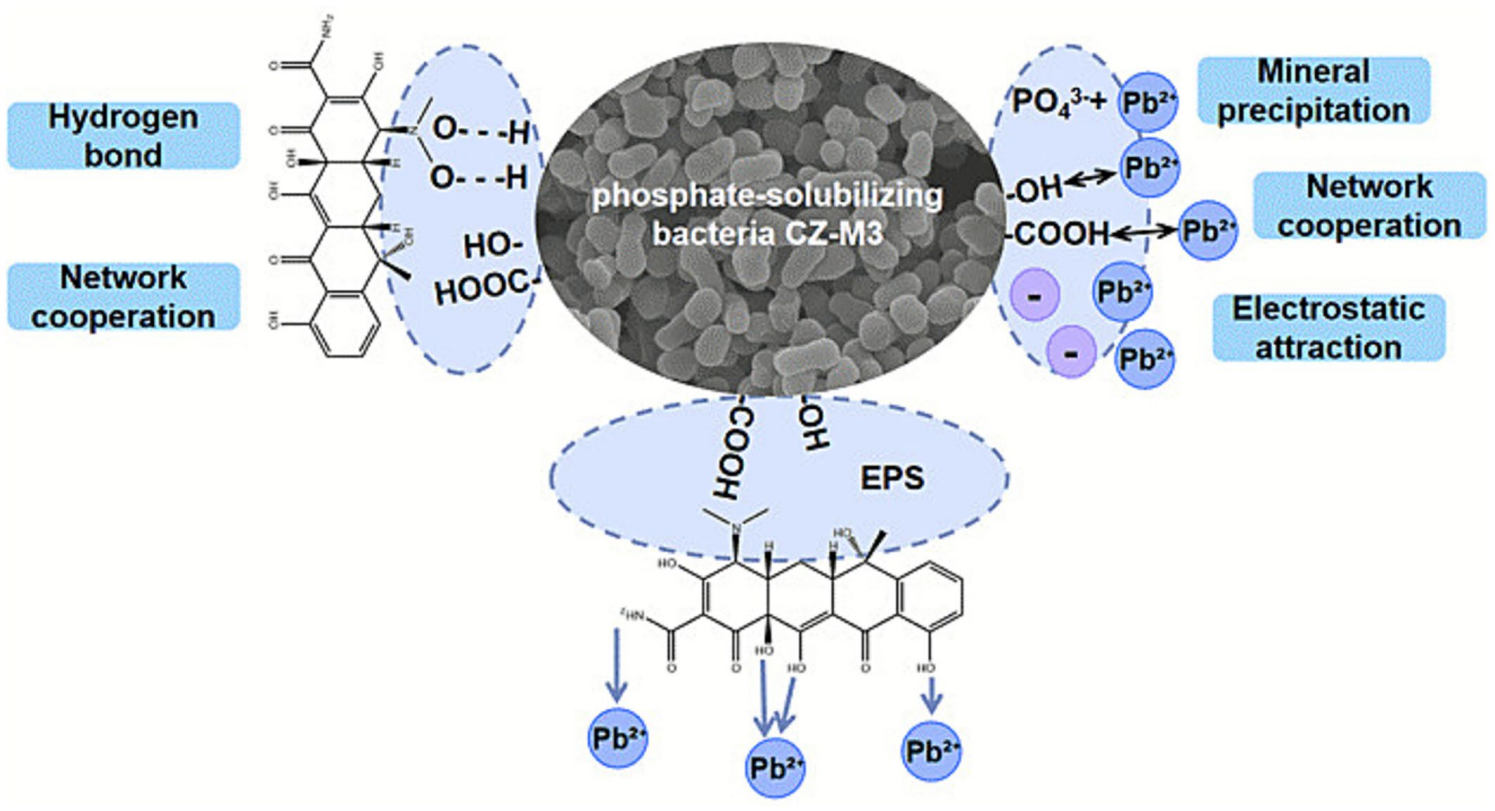
Schematic diagram of the remediation mechanism of TC-Pb co-contamination by CZ-M3.

Based on the aforementioned experimental results, the present study elucidates the principles of the multi-mechanistic synergistic action employed by the phosphate-solubilizing bacterium CZ-M3 to remediate TC-Pb co-contamination. The core mechanism involves the regulation of bacterial metabolism by the stress environment and the series of responses it elicits (Figure 7). First, phosphate precipitation is the dominant mechanism for immobilizing Pb^2+^. CZ-M3 releases PO₄^3−^ by secreting organic acids to dissolve insoluble phosphorus sources. The PO₄^3−^ then reacts with Pb^2+^ to form stable lead phosphate precipitates [such as Pb₅(PO₄)₃Cl], thereby efficiently immobilizing Pb^2+^. Second, the TC stress-induced alteration of the organic acid metabolic pathway is key to enhancing Pb immobilization. High-concentration TC stress inhibits the TCA cycle, leading to significant changes in the strain’s organic acid secretion profile, characterized by a sharp accumulation of tartaric acid. As an organic acid containing multiple carboxyl groups, tartaric acid not only promotes phosphorus dissolution but also can form stable complexes with Pb^2+^. It synergistically promotes the formation of lead phosphate precipitates by providing locally high concentrations of PO₄^3−^ and altering the interfacial environment. Furthermore, adsorption and complexation by EPS provide important supplementary mechanisms. Under co-contamination stress, CZ-M3 secretes more EPS components, such as humic acid-like substances. These EPS components, rich in functional groups like carboxyl and hydroxyl groups, can directly immobilize TC and Pb^2+^ through electrostatic adsorption and complexation, thereby enriching pollutants for subsequent precipitation reactions.

In summary, CZ-M3 achieves remediation through a core pathway and multiple synergistic mechanisms: under TC-Pb co-contamination stress, the strain actively responds through metabolic pathway alterations (tartaric acid accumulation) and increased EPS secretion. Among these, the increase in tartaric acid directly complexes Pb^2+^ on one hand, and, together with other secreted organic acids, primarily immobilizes Pb^2+^ via phosphate precipitation on the other hand. Simultaneously, adsorption by EPS and complexation by organic acids collectively contribute to TC removal. These interconnected and synergistic mechanisms constitute the unique capability of CZ-M3 for the efficient remediation of TC-Pb co-contamination.

## 4 Conclusion

This study successfully isolated a phosphate-solubilizing bacterium, *Microbacterium* sp. CZ-M3, with high potential for the remediation of TC and Pb^2+^ co-contamination. Beyond its efficient phosphate-solubilizing capacity, CZ-M3 employed a unique synergistic mechanism to achieve a high removal efficiency under high concentrations of TC and Pb^2+^ stress. Mechanistic investigations revealed that the remediation relies on a core pathway: under contaminant stress, the bacterial metabolism was reprogrammed, which was characterized by a significant shift in the secretion profile towards the accumulation of tartaric acid. This process acted in concert with increased secretion of EPS, collectively enhancing a synergistic remediation network dominated by lead phosphate precipitation and supplemented by organic acid complexation and EPS adsorption. Most importantly, this work uncovered a concentration-dependent antagonistic effect between the pollutants: a high concentration of Pb^2+^ primarily exerted biotoxicity, inhibiting bacterial activity and TC removal, whereas a high concentration of TC acted as a stress signal that activated the bacterium’s Pb^2+^ immobilization capacity, likely by regulating central metabolism (e.g., inhibiting the TCA cycle). This finding deepened our understanding of microbial adaptive responses to complex pollution stress. The elucidation of this synergistic “phosphate precipitation - organic acid complexation - EPS adsorption” mechanism deepened our understanding of microbial responses to co-contamination and provides a theoretical basis for developing advanced bioremediation strategies.

## Data Availability

The original contributions presented in the study are included in the article/supplementary material, further inquiries can be directed to the corresponding authors.
